# Role of human herpesvirus homologs of infected cell protein 27 (ICP27) in the biogenesis, processing, and maturation of mRNAs

**DOI:** 10.1128/mbio.00291-25

**Published:** 2025-03-04

**Authors:** Abel A. Soto-Machuca, Gerardo E. Ortiz, Javier Carbone-Schellman, Ignacio A. Pastén-Ferrada, Angello Retamal-Díaz, Alexis M. Kalergis, Pablo A. González

**Affiliations:** 1 Millennium Institute on Immunology and Immunotherapy, Facultad de Ciencias Biológicas, Pontificia Universidad Católica de Chile, Santiago, Chile; 2 Departamento de Biotecnología, Facultad de Ciencias del Mar y Recursos Biológicos, Universidad de Antofagasta, Antofagasta, Chile; 3 Centro de Investigación en Inmunología y Biotecnología Biomédica de Antofagasta, Hospital Clínico Universidad de Antofagasta, Universidad de Antofagasta, Antofagasta, Chile; 4 Departamento de Endocrinología, Escuela de Medicina, Pontificia Universidad Católica de Chile, Santiago, Chile; The Ohio State University, Columbus, Ohio, USA

**Keywords:** RNA transcription, RNA modification, mRNA export, mRNA translation, human herpesviruses

## Abstract

Herpesviruses are enveloped viruses with large double-stranded DNA genomes that are highly prevalent in the human population and elicit numerous types of clinical manifestations, from mild to severe. These viruses are classified into three subfamilies: *alpha*-, *beta*-, and *gammaherpesvirinae*, all capable of establishing life-long persistent infections in the host. As strict intracellular parasites, these viruses have evolved molecular determinants to support and modulate viral and host gene transcription processes during infection and the translation of messenger RNAs (mRNAs) to synthesize proteins that participate in cellular pathways promoting their replication cycles and virion formation. Notably, some of these proteins have functional RNA-binding domains consisting of arginine-glycine-glycine (RGG) amino acid (aa) sequences that, when methylated, regulate their nucleic acid-binding capacities and can influence the export of mRNAs lacking introns from the nucleus into the cytoplasm. Additional domains and motifs in these proteins mediate their interactions with regulatory proteins related to RNA splicing, either promoting or repressing mRNA processing. Notably, all human herpesviruses (HHVs) encode in their genomes proteins that share homology with infected cell protein 27 (ICP27) of herpes simplex virus type 1 (HSV-1), which can significantly impact the biogenesis of mRNAs and their processing during infection. Here, we review and discuss the roles of ICP27 and the corresponding homologs encoded in different human herpesviruses, focusing on their similarities and differences in structure and function. A more profound knowledge of the role of key viral factors required for effective herpesvirus replication could aid in the design and identification of novel antivirals to treat the diseases produced by these viruses.

## INTRODUCTION

Human herpesviruses (HHVs) are enveloped, double-stranded DNA viruses present at a high prevalence in the human population (1, 2). HHVs are classified into three subfamilies: *alpha*-, *beta*-, and *gammaherpesvirinae*. Herpes simplex viruses type 1 and type 2 (HSV-1 and HSV-2, respectively) and varicella-zoster virus (VZV) belong to the *alphaherpesvirinae* subfamily, which mainly infect skin epithelial cells and establish latency in neurons (2). However, in the case of HSV-1 and HSV-2, these viruses can also infect innate immune cells, such as dendritic cells (DCs), macrophages, and natural killer (NK) cells, among others, which are key for activating, regulating, and mediating antiviral host responses (3, 4). On the other hand, betaherpesviruses, such as the human cytomegalovirus (HCMV) and human herpesviruses 6 and 7 (HHV-6 and HHV-7, respectively), have a broad cellular tropism that includes epithelial, mesenchymal, and immune cells, and can establish latency in cells belonging to monocytic lineages (5). Lastly, Epstein-Barr virus (EBV) and Kaposi’s sarcoma-associated herpesvirus (KSHV) are gammaherpesviruses that are characterized by their capacity to infect and establish latency in B cells and T cells (6).

A prominent feature of HHVs is the presence of a lipid envelope from which numerous glycoproteins protrude (7). Between the envelope and viral capsid is a region known as the tegument, with the number and nature of the viral proteins within this structure depending on each HHV (7). For instance, in HSV-1, the tegument consists of >20 proteins that play important roles early in infection, such as evasion of cellular antiviral responses and supporting capsid migration to the nuclear membrane (8). The capsids, which have icosahedral structures, are formed by pentameric and hexameric protein complexes encompassing the viral genome (7). Once the capsids enter the cytoplasm, after the viral and cellular lipid bilayers have fused, they are carried to nuclear pores through microtubules (9). At this place, the viral genome is injected into the nucleus, where the transcription of viral genes initiates with the use of the host RNA polymerase II (RNApol II) (10–12). The transcription of viral genes in herpesviruses occurs in a cascade-type manner, with immediate-early genes (IE, or alpha) genes being transcribed immediately after infection, then early (E, or beta) genes, and finally late (L, or gamma) genes (7, 9).

A key process for the translation of viral messenger RNAs (mRNAs) is their maturation and export to the cytoplasm, which in eukaryotic cells may depend on a series of modifications occurring to the mRNA along the transcription process (13). Importantly, numerous viruses are known to modulate post-transcriptional modifications over viral and host mRNAs to favor their replication cycles (14, 15). In this regard, herpesviruses require several components of the host transcriptional machinery to promote the transcription of their genes, and they may also encode proteins that modulate the maturation and export of viral and host mRNAs to the cytoplasm (12, 16). Notably, the ICP27 protein of HSV-1 (herein ICP27) has been reported to play key roles in numerous processes related to host and viral mRNAs during infection, with some of these features extending onto ICP27 homologs in other herpesviruses. In this review, we sought to revise and discuss currently available evidence regarding the roles of ICP27 and the different ICP27-like proteins in HHVs, emphasizing their similarities and differences, both structural and functional.

## MATURATION OF mRNAs

mRNA maturation is the process by which primary mRNA transcripts are modified to acquire a form compatible with their translation (17). Recapitulating, mRNAs undergo three main structural modifications during transcription: the addition of an N^7^-methylguanosine modification at the 5′ end of the transcript (m^7^G/CAP, or CAP), the removal of introns, and the addition of a long extension of adenosine nucleotides at the 3′ end of the mRNA (17). mRNA capping (i.e., the addition of a CAP) at the 5′ of the mRNA is mediated by a set of enzymes called mRNA capping enzymes, most notably with RNA guanine 7-methyltransferase (RNMT) and cap methyltransferases 1 and 2 (CMTR1-2) playing key roles in the capping process (18). Then, mRNA splicing mediates intron excision from the transcript and exon ligation, which can be mediated by autocatalytic reactions or protein-RNA complexes (e.g., the spliceosome) (19). CAP plays a critical role in mRNA accessing the 40S ribosome subunit by recruiting host factors facilitating translation initiation. Indeed, CAP is recognized by translation initiation factors 4F and 4B (eIF4F and eIF4B, respectively), which are required to start protein translation (20). After the CAP is added to the 5′ of the mRNA during transcription, it is extended at its 3′ terminus by polyadenylation, which consists of the addition of numerous consecutive adenosines to form a poly-A sequence at its end. This process is mediated by a multi-protein complex called cleavage and polyadenylation complex (CPAC) and comprises approximately 20 individual protein subunits (21). Importantly, the polyadenylation of mRNA favors various processes related to its translation, such as mRNA export, the prevention of mRNA degradation by exonucleases, and transcript circularization to initiate translation (22). Once capping, intron removal, and polyadenylation have been carried out, mRNAs are considered mature. However, they can be further modified during the transcription process and afterward, which has been extensively reviewed by Soto et al. (1). For the export process, a multi-protein host cell structure termed the transcription-export (TREX) protein complex is considered to play a crucial role in mRNA export (23). Consistent with this notion, TREX-mutant yeasts display defects in mRNA export (24). The TREX protein complex is made of three sections: the transcription and export complex homolog sub-complex (THO), the RNA helicase Asp-Glu-X-Asp (DExD-box, with the “box” indicating the repeating structural features within the helicase domain), ATP-dependent RNA helicase UAP56 also known as DDX39B, and the RNA-binding protein ALY RNA export factor (ALYREF) (25). Functional TREX-binding domains have been reported to be encoded within ICP27 of HSV-1, which would play important roles in favoring viral mRNA export to the cytoplasm (26). Furthermore, ICP27 and most herpesviruses are known to encode an ICP27 homology domain (IHD) that displays functional ALYREF-binding sequences (27).

## DOMAINS IN ICP27 AND ICP27 HOMOLOGS

**Fig 1:**
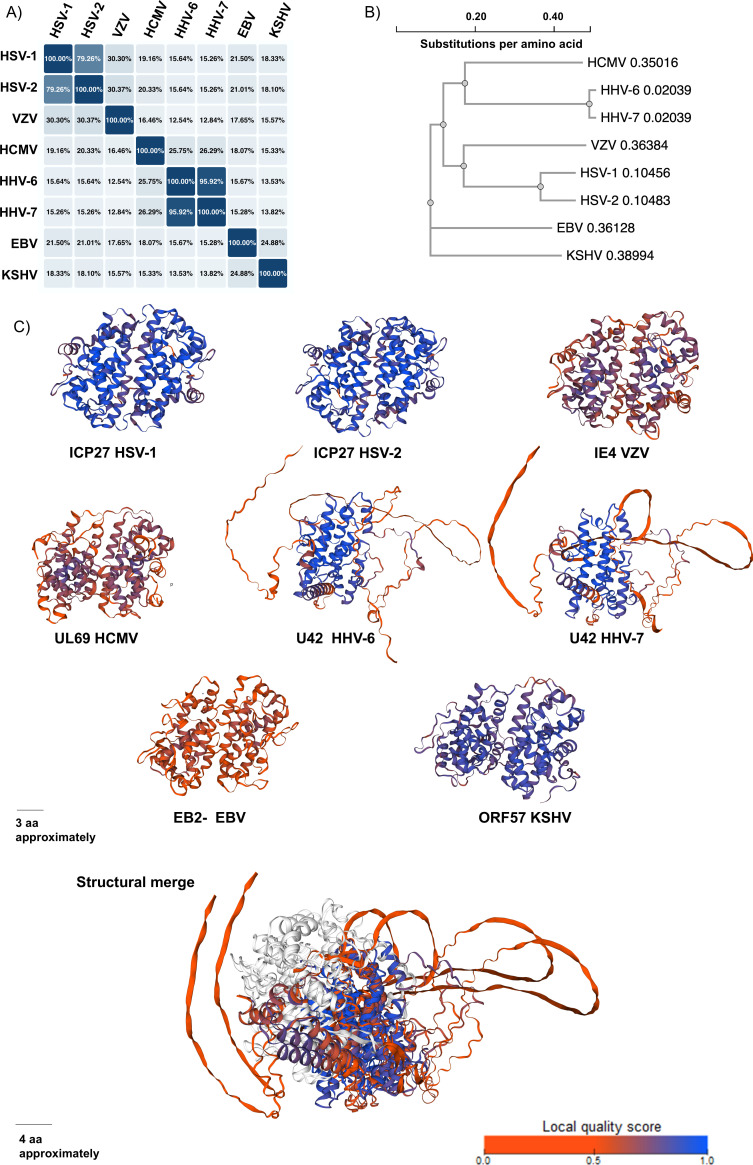
Sequence and structural homology among ICP27 homologs. (**A**) Amino acid sequence homology resulting from alignment between ICP27 of HSV-1 and each homolog. Homology percentages were obtained using the Uniprot website (https://www.uniprot.org/). (**B**) Amino acid sequences obtained in Uniprot were used to generate a phylogenetic tree in the same platform, where each branch represents the distance in substitutions per amino acids (SpA) between the different homologs, and each node represents a common ancestor. (**C**) Structural analyses of each homolog of ICP27. Amino acid sequences were obtained from Uniprot and modeled using Alphafold on its website (https://swissmodel.expasy.org/). Regions in blue describe sites with more structural confidence, and areas in red show regions with less structural confidence in the modeling process. White areas of the structural merge show regions with no structural coincidence among the different homologs. aa, amino acid.

ICP27 homologs in human herpesviruses are characterized by different degrees of sequence similarity compared to ICP27 of HSV-1, with variations occurring between 15.41% and 79.45% at the aminoacidic sequence level (Fig. 1A). Furthermore, these homologs show phylogenetic closeness based on the virus subfamily they belong to (Fig. 1B). However, despite important differences in the amino acid sequence for some ICP27 homologs compared to ICP27 of HSV-1, these proteins share nevertheless a significant degree of structural homology, with similar functional domains and a characteristic globular structure (Fig. 1C). The identification of structural similarities among these proteins has been possible thanks to the resolved molecular structure of ICP27, which was determined by cryo-electron microscopy (cryo-EM) at a 1.9 Å resolution (28).

The functional structure of ICP27 is a homodimer that adopts a cross-linked fold with a CHCC zinc finger-binding site in each monomer (Fig. 1C) (28). The dimerization of the subunits, independently confirmed by size exclusion chromatography coupled with multiangle light scattering (SEC-MALS) and analytical ultracentrifugation (AUC), is stabilized by an extensive network of intermolecular interactions and domain exchanges involving the two N-terminal helixes and the two C-terminal tails of the subunits (28). Furthermore, each ICP27 monomer contains a lid motif that can clamp the C-terminal tail to the homodimeric partner against their globular core without the formation of C-terminal secondary structure elements (28).

ICP27 of HSV-1, which is 512 aa long, has several domains that contribute to its functions within its structure. These domains, ordered from the amino- to the carboxy-terminus, are a nuclear export signal (NES) located between aa 4-16, an acidic aspartate/glutamate region with two phosphorylation sites for casein kinase II (CK2), followed by a nuclear localization signal (NLS) positioned between aa 109-138. Additionally, there is an RGG (arginine-glycine-glycine)-like RNA-binding motif between aa 137-153, an arginine-rich region (R-rich) immediately after the RGG, three K homology domains (KH1/2/3) between aa 300-512, which allow the protein to bind to RNA/ssDNA, a C-X_2_-C-X_4_-H-X_4_-C (CCHC) zinc finger domain between aa 399-487, and finally, the ICP27 homology domain located between aa242-512 (29).

Regarding ICP27 of HSV-2, the protein is 512 aa long and has 79.45% homology with ICP27 of HSV-1. The motifs in this protein are similar to those of ICP27 of HSV-1, with ICP27 of HSV-2 also encoding an NES domain located between aa 5-17, an NLS at aa 109-138, an RGG domain between aa 138-152, the IHD domain within aa 303-509, and a zinc finger domain within aa 400-488 (30). Compared to ICP27 of HSV-1, its homolog in HSV-2 lacks KH domains encoded within the aa 300-512 of ICP27 belonging to HSV-1 (30).

On the other hand, the ICP27 homolog in VZV is IE4, a 452 aa protein that shares 28.01% homology with its HSV-1 counterpart (Fig. 1A). The IE4 protein contains an NLS between aa 129-144, an NES motif between aa 320-331, and an RNA-binding domain (RBD) between aa 70-181 that is not of the RGG type (unlike other alphaherpesviruses); inside of the RBD, there are three arginine-rich regions (Ra/Rb/Rc) located between aa 70-181 (31).

Regarding HCMV, the corresponding homolog of ICP27 of HSV-1 in this virus is UL69, which is 744 aa long and has 18.95% sequence homology compared to ICP27 of HSV-1 (Fig. 1A). UL69 is likely the ICP27 homolog with the most abundant structural evidence available after ICP27 of HSV-1, as its structure was crystallized and resolved by transmission electron microscopy (TEM) and SEC-MALS at 25–50 Å of resolution (16). The structural resolution of ICP27 homologs has been paramount for a better understanding of the features and multifunctional properties of these proteins. However, these types of proteins are typically challenging to crystallize due to their structural complexity and multiple interaction sites with other molecules (28). Yet, a better understanding of the structural features of UL69 will undoubtedly shed light on its interactions with host factors, such as those related to the nuclear export of RNA, potentially contributing to the development of future HCMV-targeted therapies.

UL69 has domains that are conserved within ICP27 homologs of alphaherpesviruses, as this protein also has an NES (aa 18-47), an NLS (aa 596-625), and an arginine-rich RNA-binding domain (aa 16-140) similar to IE4 of VZV. UL69 has no RGG domain as ICP27 but has an arginine-rich RNA-binding motif (ARM). This region contains short arginine-rich and lysine-rich sequences and a TRKQAR aa sequence characteristic of this motif. The UL69 ARM is divided into R1, R2, and RS. *In vitro* and *in vivo* experiments have determined that R1, R2, and RS are critical for UL69 to bind to RNA (31, 32). Despite having distinct RNA-binding domains as ICP27 of HSV-1, UL69, as well as other ICP27 homologs, has this domain at the N-terminal end, similar to ICP27 of HSV-1 and IE4 of VZV (31).

Regarding ICP27 homologs in HHV-6 and HHV-7, these proteins named U42 in both viruses are 515 aa and 512 aa long, respectively, and share 16.09% and 15.41% amino acidic sequence with ICP27 of HSV-1 (Fig. 1A). Given the lack of structural and functional studies related to these proteins, for now, they have only been reported to possess an IHD at aa 137-361 for U42 of HHV-6 and at aa 138-362 for U42 of HHV-7 (Uniprot P52354, P52539). To emphasize that, at present, there are no reports indicating whether these proteins contain NES or NLS motifs at their N- or C-termini.

Regarding gammaherpesviruses, the ICP27 homolog EB2 in EBV is 479 aa long and shares a relatively modest homology with ICP27 of HSV-1 at the amino acidic sequence level, which is reported to be 20% (Fig. 1A). Notably, EB2 of EBV contains overlapping domains compared to the other ICP27 homologs, as it has an NLS motif at aa 126-146, an NES domain at aa 60-141, and an arginine-rich domain that does not have an RGG located between aa 189-224 (33). Finally, regarding KSHV, the ICP27 homolog is ORF57, a 455 aa protein with 19.05% homology compared to ICP27 of HSV-1 at the amino acidic sequence level (Fig. 1A). Importantly, the domains encoded within this ICP27 homolog are poorly described in the literature. Nevertheless, ORF57 is predicted to contain an NLS, an arginine-rich motif with an RGG, a leucine zipper DNA interaction domain, a hydrophobic glycine–leucine–phenylalanine–phenylalanine motif (GLFF), and an adenine-thymine (AT)-hook DNA-binding motif (34).

Taken together, all herpesviruses encode ICP27 homologs, although some show low homology at the aa sequence level with that of the ICP27 protein of HSV-1, which serves as a reference. Yet, these viral proteins display numerous conserved domains and overall share a common structure (Fig. 1B). For example, the most conserved domains among ICP27 homologs in human herpesviruses are the NES and NLS motifs, followed by the different RNA-binding domains.

## ICP27 HOMOLOGS IN VIRAL TRANSCRIPTION

All human herpesviruses must use the host transcription machinery to transcribe their genes. Notably, ICP27 of HSV-1 is one of the most studied herpesvirus proteins known to modulate this process. This protein has been described to interact with the host RNApol II’s C-terminus in its phosphorylated and non-phosphorylated forms (35). Phosphorylation of the C-terminus of the RNApol II is mediated by cyclin-dependent kinases (CDKs) and is usually associated with changes in the elongation capacity and dynamics of this enzyme, as well as the recruitment of factors required for transcription, such as those forming the pre-initiation complex (PIC) (36, 37). To interact with the RNApol II, both the C- and N-terminal ends of ICP27 have been reported to be required, as mutations in these regions affect the binding of this viral protein to the phosphorylated and non-phosphorylated forms of RNApol II in epithelial cells (HeLa cells) (35).

In addition, ICP27 has been reported to interact with viral protein 16 (VP16) of HSV-1, a well-known regulator of viral gene transcription, commonly known as a “central” regulator of this process, as it is involved in the promotion of transcription of HSV-1 immediate-early (IE) genes and is indispensable for viral replication (38). Furthermore, VP16 acts together with other host regulatory proteins, such as octamer-binding protein 1 (Oct-1) and host cell factor-1 (HCF-1), to enhance the transcription of viral genes (39). Indeed, ICP27 has been suggested to interact with the C-terminus end of the RNApol II to promote its recruitment to viral genes, specifically through interactions with components of the VP16-induced complex (VIC) (40). However, the role of this interaction, or whether it is required for VP16 to exert its transactivation role, has not been fully established.

Regarding the homolog of ICP27 in HSV-2, at present, it has not been reported whether the aa sequence differences with ICP27 of HSV-1 relate to differences in the ability of the former to interact with RNApol II or modulate viral gene transcription. This scenario is different for the ICP27 homolog IE4 of VZV, which has been reported to act as a transactivating factor for viral genes in human T lymphocyte primary cell cultures and the monkey fibroblast cell line MRC-5 (41). The only study currently available that has assessed a role for IE4 in gene transcription, reported in an epithelial cell line (HeLa cells), suggests that this viral protein can interact with the host transcription factor IIB (TFIIB), which is part of the cellular transcription initiation complex. This interaction was described to occur through an arginine-rich domain located at aa 111–181, as the deletion of this region in IE4 resulted in a loss of interaction between both proteins (42). Furthermore, the impact of the loss of the interaction between IE4 and TFIIB resulted in reduced expression of the viral thymidine kinase (TK, *UL23* gene) at the protein level (42). However, it is unclear whether the mechanism related to decreased viral TK expression in these cells was due to transcriptional downregulation or altered mRNA export to the cytoplasm of the corresponding transcript, as IE4 has also been reported to act as an RNA export regulator (further discussed below). IE4 may play a broad role in gene transcription, as transfecting a plasmid encoding the reporter chloramphenicol acetyltransferase (CAT) into an embryonic lung cell line (MRC-5 cells) and then treating the cells with increasing concentrations of IE4 resulted in increased mRNA abundance of the CAT enzyme, up to 200-fold compared to control cells. However, the mechanism by which IE4 elicited this increase in mRNA abundance remains unknown (41). It should be noted that IE4 is of great importance for viral replication because virus mutants with the gene encoding IE4 deleted from the viral genome produced a lower amount of plaque-forming units (PFUs), namely, in epithelial cells (MeWo cells) when compared to wild-type (WT) viruses (43). When evaluating whether the role of IE4 could be substituted in *trans* by its homologous protein from HSV-1, namely, ICP27, it was found that this latter protein did not substitute the role of IE4 in promoting normal virus yield. This outcome suggests that despite the existence of a high aa sequence homology between IE4 and ICP27, these proteins are not interchangeable and have distinct functions (43).

On the other hand, UL69 of HCMV has also been characterized as a transactivation factor, with this function being mediated either through the direct interaction of this protein with DNA or by facilitating the recruitment of proteins essential for gene transcription. Experiments consisting of transient expression of UL69 showed that when transfecting a plasmid encoding the HCMV phosphoproteins p34, p43, p50, and p84 (early gene *UL112*) with a luciferase reporter, there was at least a 10-fold increase in luciferase activity compared to controls in primary human foreskin fibroblasts (HFF cells). However, the observed effect was not visible for the late gene *UL86* (44). The same study also explored whether the reported effect was exclusively related to genes of this particular virus or affected other viral or host determinants. It was observed that other constructs containing the *UL23* gene (TK protein) of HSV-1, the long terminal repeat (LTR) sequence of the Rous sarcoma virus (RSV), the LTR sequence of the human immunodeficiency virus (HIV), and the cellular promoters of beta-actin and phosphoglycerol kinase (PGK) had transcript levels of all these genes increased in the presence of UL69 in HFF cells (44). However, the mechanism through which UL69 stimulated the transcription of these genes has not been reported (32). In this same line, it has been determined that UL69 can interact indirectly with RNApol II to promote viral gene transcription through histone chaperone and transcriptional elongation factor 6 (SPT6) and that this interaction is mediated by aa in the ICP27 HD domain and within the NES sequence (45). However, the effects of the interaction between ICP27-SPT6-RNA Pol II have not been studied, nor whether this interaction is critical for recruiting RNApol II to gene promoters.

Regarding the ICP27 homolog proteins in HHV-6 and HHV-7, to date, they have not been reported to play any role in promoting viral gene transcription or functions related to mRNA biogenesis, likely because they have not yet been studied. Thus, these factors are interesting determinants to be analyzed to assess their impact during infection by these viruses and determine whether there are common features that can be generalized among ICP27 homologs. Thus, further studies are needed on these viral factors.

Finally, regarding the gammaherpesviruses EBV and KSHV, the EB2 and ORF57 ICP27 homologs, respectively, have been extensively studied in the context of functions such as splicing and mRNA export (Table 1), yet none so far related to relevant roles in viral gene transcription.

**TABLE 1:** Role of ICP27 homologs in transcriptional and post-transcriptional mRNA processing

Virus	ICP27 homolog	Homology with ICP27 of HSV-1 (%)	Encoding gene	Effects over the processing of host mRNAs	Effects over the processing of viral mRNAs	Cell type/cell line	References
Herpes simplex virus type 1 (HSV-1)	ICP27	N/A	*UL54*	Blocks the transcription of host genes and impairs host mRNA translation by interacting with the cleavage and polyadenylation specificity factor (CPSF) protein. Inhibits host mRNA splicing.	Interacts directly with host export factors ALYREF (an export adapter factor involved in nuclear export of spliced and unspliced mRNA) and nuclear export factor 1 (NXF1) to export viral mRNAs to the cytoplasm.	Human foreskin fibroblasts (HFFs), epithelial cells (HeLa cells), and rabbit skin fibroblasts (RSFs).	(46, 47)
Herpes simplex virus type 2 (HSV-2)	ICP27	79.45	*UL54*	Blocks the transcription of host genes and impairs host mRNA translation by interacting with the cleavage and polyadenylation specificity factor (CPSF) protein. Inhibits host mRNA splicing.	Interacts directly with host export factors ALYREF (an export adapter factor involved in nuclear export of spliced and unspliced mRNA) and nuclear export factor 1 (NXF1) to export viral mRNAs to the cytoplasm.	Human foreskin fibroblasts (HFFs), epithelial cells (HeLa cells), and rabbit skin fibroblasts (RSFs).	(46, 47)
Varicella-zoster virus (VZV)	IE4	28.01	*ORF4*	None reported.	Interacts with auxiliary export factors (serine/arginine-rich splicing factor 3, SRSF3; nuclear export factor 1, NXF1; and ALYREF [an export adapter factor involved in the nuclear export of spliced and unspliced mRNA]) to export viral mRNAs to the cytoplasm.	Human melanoma cells (MeWo) and epithelial cells (HeLa cells).	(32, 41, 42, 48)
Human cytomegalovirus (HCMV)	UL69	18.95	*UL69*	Disrupts host mRNA processing by interacting with cellular factors, such as UAP56 and URH49, interfering with mRNA export and splicing while altering host mRNA translation.	Promotes viral gene expression by enhancing transcription and facilitating nuclear export of intronless viral mRNAs via interactions with U2AF65-associated protein 56 (UAP56) and UAP56-related helicase 49 (URH49). Boosts viral protein synthesis through UAP56 interactions with host translation factors.	Epithelial cells (HeLa cells), Swiss mouse 3T3 cells (3T3), and human foreskin fibroblasts (HFFs).	(16, 49)
Human herpesvirus 6 (HHV-6)	U42	16.09	*U42*	None reported.	None reported.	None reported.	—
Human herpesvirus 7 (HHV-7)	U42	15.41	*U42*	None reported.	None reported.	None reported.	—
Epstein-Barr virus (EBV)	EB2	20	*BSLF2/BMLF1*	Interacts with serine and arginine-rich splicing factor 3 (SRp20). Produces alternative splicing of the host mRNA of STAT1 dysregulating STAT1 expression.	Promotes nuclear export and cytoplasmic accumulation of intronless early and late viral mRNAs through N-terminal nuclear export signal interaction with nuclear export factor 1 (NXF1) complex.	Epithelial cells (HeLa cells), human embryonic kidney cells (HEK293 cells), human embryonic kidney cells with the large T antigen of SV40 (HEK293T cells), B cells, and immortalized B cells (DG75 cells).	(50–52)
Kaposi’s sarcoma- associated herpesvirus (KSHV)	ORF57	19.05	*ORF57*	None reported.	Stabilizes viral mRNAs by interacting with ORF57 response elements (ORE), recruiting polyadenylate-binding protein 1 (PABPC-1), preventing hMTR4 degradation, and increasing export and translation efficiency through the ALYREF (an export adapter factor involved in the nuclear export of spliced and unspliced mRNAs) and the exon-junction complex (EJC).	Human embryonic kidney cells with the integration of E1 gene (293A-TOA cells), Dox-inducible RTA-expressing Kaposi’s sarcoma-derived cell line (iSLK cells), human embryonic kidney cells with the large T antigen of SV40 (HEK-293T cells), human embryonic kidney cells with the large T antigen of SV40 and transfected with BAC36 that contains the entire genome of KSHV (293T-BAC36 cells), and body-cavity-based lymphoma (BCBL-1 cells).	(34, 53–56)

In conclusion, numerous herpesvirus ICP27 homologs are known to participate in transcriptional regulation processes, with some also displaying roles in mRNA splicing and export (further discussed below), and thus deserve attention as these effects highlight these molecular determinants as potential pharmacological targets for modulating or controlling viral replication. Indeed, evidence suggests a consistent role for ICP27 homologs in alphaherpesviruses around the recruitment of RNApol II toward viral genes for promoting their transcription, particularly by interacting with proteins belonging to the host transcription initiation complex. Regarding betaherpesviruses, the role of ICP27 homologs has been extensively studied, and they have been determined to be key for transcription, acting as transactivating factors for the expression of viral genes. Furthermore, UL69 is key for HCMV effective replication, as it enhances the transcription and processing of viral mRNAs and works closely with the host’s RNA export machinery to promote viral gene transcription and proper translocation to the cytoplasm. Regarding gammaherpesviruses, remarkably, to date, these have not been linked to any role in viral gene transcription.

## ICP27 HOMOLOGS AND THEIR ROLES IN RNA SPLICING

Current evidence suggests that HSV-1 has few genes spliced during infection, with most genes encoded within its genome being devoid of introns (intronless) (57). However, ICP27 may act as a host and viral mRNA splicing regulator. Indeed, current evidence shows that ICP27 physically interacts with spliceosome proteins, such as U1 snRNP, through its C-terminal domain (58). Regarding mRNA splicing processes mediated by the host cell, an accumulation of host pre-mRNA transcripts has been observed in the presence of ICP27, and a lack of expression of this protein, such as during infection with an ∆ICP27 mutant virus, reverts this condition, suggesting that ICP27 is associated with an inhibition of pre-mRNA splicing (46). Consistently, inhibiting splicing in infected cells translates into pre-mRNA accumulation with introns, which has been reported in other studies up to 8 hours post-infection (57). On the other hand, additional ICP27 domains, such as the RGG domain located in the N-terminus, can interact with other splicing factors, such as serine and arginine-rich splicing factors (SRSF) SRSF-1, SRSF-2, SRSF-3, and SRSF-7 (59). Depletion of SRSF2 was shown to impair viral replication, highlighting its involvement in viral RNA biogenesis and eventual splicing of viral transcripts (60). Additionally, the RNA-binding domain of ICP27 has been reported to interact with serine/arginine protein kinase-1 (SRPK1), and current evidence suggests that ICP27 can reduce SRPK1 activity due to a high-affinity interaction between both proteins. This effect elicits the hypophosphorylation of SRPK1 and negatively affects its activity as a splicing regulator, leading to its impaired function during spliceosome assembly and evidencing a direct mechanism through which ICP27 can alter splicing (58, 59).

**Fig 2:**
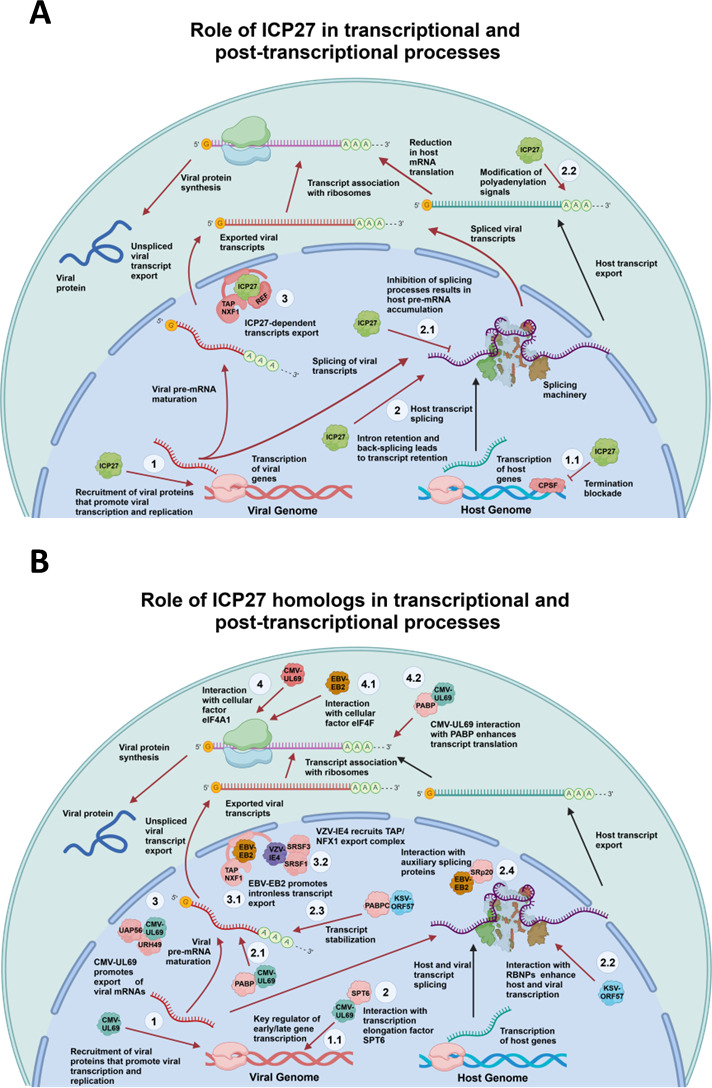
Role of HSV-1 ICP27 and ICP27 homologs in mRNA biogenesis. (**A**) Role of the HSV viral protein ICP27 (light green) in transcriptional and post-transcriptional processing of mRNAs. ICP27 plays various roles that favor the transcription of viral genes over host genes, the maturation of viral transcripts, the inhibition of host gene splicing (resulting in intron retention and aberrant transcripts), and the promotion of viral transcript export to the cytoplasm, and modify polyadenylation signals of host transcripts. (1) ICP27 recruits viral proteins participating in viral gene transcription and replication. (1.1) ICP27 blocks transcription termination of viral genes by interacting with the CPSF protein, thereby blocking mRNA 3′ cleavage. (2) ICP27 retains introns and induces back-splicing in host transcripts, leading to nuclear retention. (2.1) ICP27 blocks host transcript splicing, leading to pre-mRNA accumulation. (2.2) ICP27 induces polyadenylation signals in host transcripts, affecting their translation. (3) ICP27 interacts with the host export complex ALYREF to enhance viral transcript export to the cytoplasm. (**B**) Role of ICP27 homologs in transcriptional and post-transcriptional processing of mRNAs. VZV-IE4 (purple), HCMV-UL69 (calypso), EBV-EB2 (orange), and KSHV-ORF57 (light blue) roles in transcriptional and post-transcriptional processing of mRNAs that favor viral replication. (1) HCMV-UL69 recruits viral proteins that participate in viral gene transcription and the replication of HCMV. (1.1) HCMV-UL69 plays important roles in the transcription of early and late HCMV genes. (2) HCMV-UL69 interacts with the host transcriptional elongation factor SPT6. (2.1) HCMV-UL69 also interacts with host poly(A)-binding protein (PABP). (2.2) KSHV-ORF57 enhances viral and host transcript splicing. (2.3) KSHV-ORF57 interacts with polyadenylate-binding protein cytoplasmic (PABPC) and facilitates viral transcript stabilization. (2.4) EBV-EB2 interacts with the host splicing factor Srp20 and induces the alternative splicing of STAT1, reducing the corresponding transcript isoforms. (3) HCMV-UL69 interacts with export factors UAP56 and URH49 to promote viral gene transcript export to the cytoplasm. (3.1) EBV-EB2 promotes intron-less transcript export to the cytoplasm. (3.2) VZV-IE4 recruits the host TAP/NFX export complex to stimulate viral gene transcript export to the cytoplasm. (4) HCMV-UL69 interacts with the cellular factor eIF4A1, promoting viral gene transcript translation. (4.1) EBV-EB2 interacts with cellular factor eIF4F to promote viral gene transcript translation. (4.2) HCMV-UL69 interacts with PABP to enhance viral transcript translation. Red arrows indicate viral effects/processes, while black arrows indicate host cell effects/processes. Pink proteins represent host proteins. VZV, varicella-zoster virus; HCMV, human cytomegalovirus; EBV, Epstein-Barr virus; KSHV, Kaposi sarcoma herpesvirus.

Although ICP27 has been shown to interact with intronless viral mRNAs, it has also been reported to be unable to interact with spliced viral mRNAs that have already undergone splicing (61). Why and how ICP27 interacts only with viral mRNAs that have already undergone splicing is currently unknown and is an important question to be addressed in future studies. Although HSV-1 infection *per se* inhibits host mRNA splicing through the function of ICP27, this process can also elicit the generation of novel host transcripts by a mechanism that involves alternative transcription termination, resulting in novel splicing sites that can be host specific (57). Finally, another mechanism by which ICP27 can modulate the splicing of host mRNAs is by modifying cryptic polyadenylation signal sequences (PAS) located within introns in 5′ regions of the transcripts (Fig. 2A) (29). These cryptic sequences can generate the retention of introns within mRNAs, thus negatively affecting their translation if the introns are present in regulatory sequences, such as the 5′ untranslated region (5′ UTR), because they can affect the binding and interactions of these mRNAs with translation-related proteins (62). Furthermore, these novel transcripts can contain premature terminal codons (PTCs), which may promote their degradation by host mRNA quality control processes, such as those mediated by the nonsense-mediated decay system (NMD) (62).

One interesting but not widely studied role of ICP27 is its involvement in facilitating back-splicing. This type of splicing differs from canonical splicing because it does not produce a linear mRNA but rather a circular RNA product (63). Previous reports have described that ICP27 is crucial for enabling the back-splicing of the long noncoding RNA called nuclear paraspeckle assembly transcript 1 (NEAT-1) in fibroblasts (64). However, it remains to be determined whether this effect, promoted by ICP27, is mediated directly or indirectly by this protein and which domains of the protein participate in this process (63).

Similar to ICP27, there is also evidence that IE4 of VZV interacts directly with splicing regulatory proteins (SRp), SRp20, serine-arginine splicing factor 7 (SRSF7), and with splicing factor 2 (SF2), although its leading interacting partner is suggested to be serine-arginine protein kinase 1 (SRPK1), which phosphorylates IE4 at Ser136 (31, 65). Furthermore, the phosphorylation of IE4 in its arginine-rich region, known as Rb, which is located between aa 112-143, has been reported to stabilize the binding of IE4 to different mRNAs. However, to date, there are no studies assessing the global impact of the interaction of IE4 with splicing regulatory proteins such as SRPK1 or whether IE4 has any effect on spliceosome formation that could ultimately have a negative impact on the maturation of host transcripts (Fig. 2B).

Regarding betaherpesvirus ICP27 homologs, there is little evidence supporting a role for these proteins in regulating splicing. This is mainly because no functional studies involving U42 of HHV-6 or HHV-7 are available. However, there is evidence of an interaction between the ICP27 homolog UL69 of HCMV with the DEAD box family RNA helicase UAP56, a splicing regulator. Indeed, UL69 interacts with the U2 snRNP complex to mediate its entry into the spliceosome as evidenced in epithelial cells (HeLa cells) (66). This interaction was found to occur between a 12-aa region located near the N-terminus of UL69 with UAP56 and URH49 (67), yet this interaction has not been reported to have either negative or positive impacts on host splicing but instead plays a role in mRNA export (Table 1) (67). In epithelial cells (HeLa cells), the interaction between UL69 and UAP56 suggests that the former may influence splicing to enhance viral mRNA processing by binding to the latter and affecting the role of the U2 snRNP complex during splicing, ensuring viral transcript processing required for HCMV replication (Fig. 2B). This reported interaction showcases the ability of UL69 to exploit the host machinery for both mRNA export and splicing, highlighting its multifunctional capabilities. Understanding the mechanism by which UL69 exerts its effect on UAP56 could open promising venues for novel antiviral targets (67).

Regarding the HHV-6 and HHV-7 homologs of ICP27, namely, U42, these proteins seemingly have not been studied in splicing, with a profound lack of functional studies regarding these proteins in general. Contrarily, the gammaherpesvirus ICP27 homolog EB2 of EBV has been reported to play a fundamental role in the cytoplasmic accumulation of unspliced mRNAs when these derive from transcript precursors that have weak cryptic 5′ splicing sites (50). Conversely, EB2 does not impact the export of mRNAs whose precursors have canonical splicing sites. Considering that the EBV genome contains subsets of early and late intronless genes, this mechanism will likely confer an advantage to the virus for preferentially exporting viral mRNAs to the cytoplasm (51, 68). An interesting role of EB2 is its ability to interact with auxiliary splicing proteins, generating non-functional isoforms of mRNAs that possibly favor viral replication (69). For example, EB2 participates in the alternative splicing of the mRNA of signal transducer and activator of transcription-1 (STAT1) by interacting with the host SRp20 splicing factor and leading to an overall decrease in the corresponding protein isoforms STAT1a and STAT1b, and an inhibition of apoptosis related to gamma interferon (IFN-γ), a type-II interferon. This would benefit EBV replication by inhibiting cell host defense mechanisms (70). Unlike other herpesvirus ICP27 homologs that play relevant roles in modulating host gene splicing, in EBV, this process seems to be mediated separately by other viral proteins, namely, the Epstein-Barr nuclear antigen 2 (EBNA 2) and the Epstein-Barr nuclear antigen leader protein (EBNA-LP). Both of these EBV proteins have RGG-like RNA-binding sites and are reported to interact and modulate the function of RNA-binding motif 4 (RBM4), which is a splicing factor implicated in regulating alternative splicing events and is present in the nucleus, where it interacts with pre-mRNA to influence the selection of splice sites during the maturation of mRNAs (71). This interaction generates alternative splicing of host transcripts and has been linked to proinflammatory processes, such as those mediated by the NUMB endocytic adaptor protein (NUMB), which plays a critical role in various cellular processes, particularly regarding cell fate determination, endocytosis, signaling pathways, and B cell lymphoma extra-large (BCL-XL) signaling, which inhibits apoptosis (71). In primary B cells, the modulation of splicing processes in the context of EBV infection has been reported to generate novel transcripts from the host genome that are recognized by the NMD system. Thus, host transcripts that do not pass quality controls due to deficient splicing events will be degraded within the cells (Table 1) (Fig. 2B) (71, 72). Consistently, in primary B cells, novel host transcripts generated by alternative splicing processes in the context of EBV infection have been reported to be recognized and degraded by the NMD system (Table 1) (Fig. 2B) (72).

Regarding the ICP27 homolog ORF57 of KSHV, this factor has also been reported to be related to the splicing of viral transcripts (73). ORF57 has two isoforms generated by alternative splicing in lytically infected B cells, in which isoform 1 is predominant (74). Furthermore, ORF57 can form ribonucleoprotein complexes with intronless mRNAs and a diverse set of viral and host proteins, such as splicing factors. For example, ORF57 has been reported to interact with spliceosome small nuclear RNAs (snRNAs), namely, snRNAs U1, U2, U4, U5, and U6, and with the splicing factor pre-mRNA splicing factor SF2 (SF2/ASF) (34, 73). Overall, ORF57 promotes viral gene splicing, and current evidence suggests that this protein regulates the splicing of the mRNA transcript of K8, an important viral protein related to viral DNA replication (53). This splicing event was described to be possible, thanks to an interaction between ORF57 and serine/arginine-rich splicing factor 3 (SRSF3) (73), namely, by the N-terminal domain of ORF57, which was found to bind to the RNA recognition motif of SRSF3 that impaired its association with the K8b intron, which promotes its splicing in body-cavity-based lymphoma cells (BCBL-1 cell line) (73). This positive effect was observed with viral pre-mRNAs and non-viral mRNAs using a plasmid encoding beta-globin, which usually undergoes poor splicing processes due to a long exon upstream of a spliced intron (47). Thus, the observed effects for ORF57 over splicing are not only exclusive to viral transcripts but also apply to host pre-mRNAs (34, 54).

Taken together, the interaction between different homologs of ICP27 with proteins belonging to the host spliceosome machinery, such as splicing regulatory proteins (SR proteins), can elicit a wide range of splicing events, such as intron retention or exon skipping, directly affecting the function of the resulting translated proteins and impacting the expression of host genes directly. Because human herpesvirus genes are characterized mostly by being intronless, these transcripts are independent of these alterations, which likely favors their replication. In the case of ICP27 of HSV-1 and ORF57 of KSHV, these proteins can significantly impact splicing, generating alternative splicing events in host mRNAs that result in non-functional isoforms that may be degraded by mRNA quality control mechanisms, such as the NMD pathway (Table 1) (Fig. 2B). An interesting future perspective for a potentially new therapeutic will be to investigate which domains of the ICP27 homologs are critical for regulating the alternative splicing events described above in infected cells so that they can be specifically targeted with drugs.

## ICP27 HOMOLOGS AND THEIR ROLES IN mRNA EXPORT

As indicated above, most HHV transcripts are intronless, which is likely an advantage for viral mRNAs over host transcripts because the former does not require extensive processing and, therefore, can be transferred directly through the mRNA export machinery into the cytoplasm (47, 75, 76).

One of the most common roles that have emerged from ICP27 homologs is, in general, their capacity to modulate the export of mRNAs from the nucleus to the cytoplasm. As discussed earlier, ICP27 of HSV-1 contains regions that allow it to interact directly with host export factors, such as ALYREF and TAP/NXF1, which are host factors related to mRNA export. Consistently, silencing of ICP27 reduces viral mRNA export to the cytoplasm in epithelial cells (HeLa cells) (76). Furthermore, ICP27 has been proposed to potentially act as an adaptor protein for the export of viral mRNAs through its interaction with the ALYREF protein, with this interaction being given by three critical aa within ICP27, namely, W105, R107, and L108 (47). Experiments with HeLa cells infected with an HSV-1 virus containing a mutation in ICP27 that binds the ALYREF motif (WRL mutant) evidenced an accumulation of viral poly-A mRNAs in the nucleus compared to control cells. However, this accumulation was not reflected in a drastic decrease in viral titers generated from cells infected with this mutant virus, as viral yield only decreased 10-fold, suggesting that viral mRNAs likely also use other export mechanisms to reach the cytoplasm for translation (47). Regarding the interaction of ICP27 with the TAP/NXF1 export complex, some reports suggest that this interaction is critical for ICP27 for its translocation between the cytoplasm and the nucleus, as a mutant HSV-1 encoding an ICP27 protein that is unable to interact with TAP/NXF1, had ICP27 retained within the nucleus directly affecting its function. Furthermore, in this scenario, most viral transcripts were retained in the nucleus compared to cells infected with the wild-type virus (26).

IE4 of VZV also plays a role in mRNA export, which is mediated by a cysteine-rich motif in its C-terminal domain. Consistently, mutagenesis experiments in which this motif was removed from ICP27 elicited a significant reduction in the export of viral mRNAs to the cytoplasm (48). Although the mechanism by which mRNA export was affected in the absence of the C-terminal domain of IE4 remains to be determined, it is believed that this region may be involved in the dimerization of IE4, as existing studies indicate that this protein dimerizes through its C-terminal domain (31). Another motif of importance for the role of IE4 in mRNA export from the nucleus is its R-rich region within aa 70–181 (31, 65). The phosphorylation of the R-rich region of IE4, known as Rb, has been reported to stabilize the binding of IE4 to intronless mRNAs, and this post-translational modification also allowed the interaction of IE4 with the mRNA export factors TAP/NXF1 and ALYREF, facilitating viral mRNA export (31).

Like other ICP27 homologs, UL69 of HCMV is also believed to play a role in modulating the export of intronless viral mRNAs from the nucleus to the cytoplasm within infected cells (32). Consistently, UL69 has been shown to interact with cellular mRNA export proteins such as U2AF65-associated protein 56 (UAP56) and its closely related counterpart UAP56-related helicase 49 (URH49) (67). UAP56 and URH49 are classified as DEAD-box helicases, acting as RNA-dependent ATPases pivotal in bridging pre-mRNA splicing with mature mRNA export processes (77, 78). The ability of UL69 to bind to UAP56 and/or URH49 was considered critical for its ability to promote the export of viral transcripts during infection and consistently played a critical role in controlling viral replication (79). Additionally, UL69 forms a complex with the cellular helicase UAP56, which plays roles in transcript elongation and host mRNA export (49, 80, 81). UL69 also features arginine-rich motifs that directly engage in mRNA interactions, although these interactions are not indispensable for the nuclear export of transcripts. Indeed, an RNA-binding-deficient UL69 HCMV mutant allowed a cytoplasmic accumulation of an unspliced CAT reporter RNA, suggesting that, unlike its homologs, RNA binding is not essential for UL69-mediated nuclear mRNA export (82). Moreover, UL69 was found to possess nuclear export and localization sequences between aa 18–47 for the NES and 596–625 for the NLS, facilitating the translocation of this protein between the nucleus and cytoplasm. In the nucleus, UL69 was found to be primarily involved in promoting the export of viral mRNAs, while in the cytoplasm, it was related to the regulation of their translation and interaction with cellular proteins, such as DExD-box helicase 39B (DDX39B/UAP56) and DExD-box helicase 39A (DDX39A/URH49), which are located in the nucleus and cytoplasm, are part of the RNA-exporting complex, and affect these processes (80). Overall, UL69 has been reported to help the recruitment of factors mentioned above to viral mRNAs and facilitate their export from the nucleus to the cytoplasm, with UL69 acting as a mediator in this process and related to ATP-dependent helicases participating in mRNA export (79). Finally, methylation of the R1 motif of UL69 by protein arginine methyltransferase 6 (PRMT-6) has been reported to be necessary for exporting viral mRNAs to the cytoplasm and, thus, key for viral replication. Such methylations were related to changes in the secondary structure of UL69 that allowed its interaction with export-related proteins, such as UAP56, which enhances the export of viral mRNAs, thus aiding in the replication cycle of HCMV (45). Regarding the ICP27 homologs U42 of HHV-6 and HHV-7, again, at present, there is no evidence of a role for these proteins in mRNA export. However, given the elevated functional conservation of mRNA export functions in different ICP27 homologs, this function may also be conserved in these proteins, although this remains to be determined experimentally. Thus, functional studies with U42 are needed to deepen our understanding of their roles during infection and the possibility of the generalization of this function for all herpesvirus ICP27 homologs.

Notably, like other ICP27 homologs, the EB2 protein of EBV has also been described as interacting with mRNAs and relating to the accumulation of host pre-mRNAs in the nucleus that have not been fully processed (33). This role of EB2 is crucial for EBV replication because the viral genome contains very few introns. Unlike host mRNAs, which remain in the nucleus until fully processed, intronless viral mRNAs are preferentially exported to the cytoplasm. Thus, viral transcripts are likely poorly affected by the abovementioned effect elicited by the EB2 protein and could provide viral mRNAs export priority over other mRNAs, thereby facilitating viral protein production (33). Additionally, this process is supported by the N-terminal region of EB2 (71), which contains an NES that interacts with the TAP/NXF1 protein complex, facilitating the export of viral mRNAs (83). While EB2, unlike other ICP27 homologs, lacks an ALYREF domain to facilitate viral mRNA export, it has been reported nevertheless to interact with different host RNA export factors, such as TAP/NXF1, to promote the export of viral mRNAs (Table 1) (Fig. 2B).

Regarding ORF57 of KSHV, this viral factor has also been linked to roles related to the export of viral mRNAs from the nucleus to the cytoplasm. For instance, ORF57 interacts with the host export factor ALYREF and is also known to promote the recruitment of this export factor to intronless mRNAs (52). Another study reported that ORF57 can also interact with other export factors, such as the nuclear protein UAP56 interacting factor (UIF), which is a protein involved in the recruitment of mRNA export proteins, as NXF1 to the mRNA, facilitating its export through the nuclear pore (34). A main difference between UIF and ALYREF mRNA export is that ALYREF depends on splicing and the UAP56 protein, while UIF is loaded onto mRNAs via the histone chaperone protein that facilitates chromatin transcription (FACT) (52, 77). Notably, it was shown that ORF57 preferentially binds to ALYREF rather than UIF in B cells (BCBL-1 cell line), and the depletion of ALYREF generated a modest reduction in viral mRNA export, indicating that there are seemingly redundant export mechanisms for KSHV intronless mRNAs, similar to what was discussed above for HSV-1, VZV, HCMV, and EBV (78). However, the recruitment of human transcription factors, as well as the mRNA export complex, human-transcription export complex (hTREX), and the NXF1 protein, was mediated by the interaction of ORF57 with UIF (52). Taken together, the findings described to date indicate that ORF57 can mediate the export of viral mRNAs by interacting with various host export factors, although its participation in mRNA export seems not to be critical for this process (Fig. 1B).

Overall, herpesvirus ICP27 homologs share conserved functions regarding mRNA export from the nucleus to the cytoplasm. This is possible, thanks to the conserved domains present in the structure of each ICP27 homolog, such as the ALYREF motif, which allows interactions with export proteins, with the most common being the host factor NXF1, part of the hTREX export protein complex. Importantly, ICP27 homologs are likely to promote a preferential export of viral mRNAs over host mRNAs and confer themselves an advantage for the translation of viral determinants, mainly because herpesvirus pre-mRNAs are mostly intronless and therefore are somewhat immediately available for their export to the cytoplasm, unlike most host pre-mRNAs.

## ICP27 HOMOLOGS AND THEIR ROLES IN mRNA STABILITY AND TRANSLATION

One mechanism by which ICP27 of HSV-1 stimulates the translation of viral mRNAs is through its interaction with proteins linked to the translation process. For instance, it has been reported in a mouse fibroblast cell line (BHK C13) that ICP27 promotes the recruitment of the poly-A-binding protein (PABP) to mRNAs, which is responsible for transcript circularization for translation, as it has a positive impact on the stability and translational efficiency of such mRNAs (55). Indeed, in one study, ICP27-dependent recruitment of PABP was shown, which promoted the recruitment of the small ribosomal subunit through eukaryotic translation initiation factor 4G (eIF4G), a subunit of eIF4F, to initiate the translation of viral mRNAs (55). On the other hand, an interaction between ICP27 and polyribosomes has been reported within fibroblasts (BHK cells) *in vitro* and *Xenopus* oocytes *in vivo*, with the results suggesting that the C-terminal of ICP27 is required for the stimulation of translation (Fig. 2A) (84). Moreover, ICP27 has been reported to increase the translation of late viral gene mRNAs, such as that encoding infected cell protein 5 (ICP5), which is the major capsid protein of this virus. This was evidenced by the fact that mutants of ICP27 significantly affected the abundance of ICP5 in infected cells compared to cells treated with the WT virus in epithelial cells (Vero cells) (85). Furthermore, it was determined that this decrease in viral protein levels was not due to problems related to the export of viral mRNAs to the cytoplasm but rather a particular capacity of ICP27 to modulate the translation of the mRNA of ICP5, which was studied using radioactive-labeled methionine. In this experiment, a decrease in the radioactive tag of ICP5 was observed with the ICP27 mutant virus compared to the control virus, which was likely related to reduced mRNA stability and/or mRNA translation (86). Another gene with decreased expression during infection with the ICP27 mutant virus and determined in the same study was VP16. Although it was found that these reported effects of ICP27 were dependent on its C-terminus, the mechanism by which this region mediated the increased translation of these late viral genes was not resolved (85).

Regarding gammaherpesviruses, EB2 of EBV has also been associated with enhanced translational efficiency of viral mRNAs. It has been reported that EB2, as well as ICP27 of HSV-1, can promote the recruitment of the PABP protein, which in turn elicits the recruitment of eIF4G to promote the recruitment of the small ribosomal subunit to facilitate the translation of viral mRNAs. This highlights the association between protein structure and function, as EB2 only possesses 21.5% aa sequence homology with ICP27 of HSV-1 (Fig. 1A), yet they both have similar structural folds (Fig. 1C). Moreover, this interaction between EB2 and eIF4G-PABP decreased the overall sensitivity of the cells to drugs that inhibit the translation process, such as hippuristanol and L-protease X, thus leading to enhanced translation of viral mRNAs (87). In addition, EB2 has been related to promoting the translation of mRNAs derived from intronless viral genes without affecting the translation of cellular mRNAs. Notably, this effect could be reversed by adding an intron into the viral mRNA, evidencing the specificity of this viral factor for intronless mRNAs, although the mechanism related to this phenomenon remains unclear (Table 1) (Fig. 2B) (68).

The ICP27 homolog ORF57 of KSHV has also been reported to have a relevant role in promoting viral mRNA translation by regulating RNA stability. Indeed, it has been suggested that ORF57 may have domains that favor interactions with target mRNAs that possess an ORF57 response element (ORE), which elicits the recruitment of the polyadenylate-binding protein 1 (PABPC-1) that interacts with the poly-A tail of ORF57 target mRNAs. However, this phenomenon has only been studied so far with the mRNA of the viral gene encoding polyadenylated nuclear RNA (PAN), for which increased stability was observed, and thus, a similar effect involving PABPC-1 over other viral mRNAs or host mRNA remains to be determined (34). However, other mRNA-stabilizing effects mediated by ORF57 have been reported, with this protein preventing viral mRNAs from being degraded by RNA-related decay pathways, such as the nuclear exosome decay pathway (NEDP). Indeed, viral mRNA degradation can be induced by the recruitment of ATP-dependent RNA helicase DOB1 (hMTR4), which, apart from its role as a helicase, also plays an active role in selecting mRNAs for degradation (86). Interestingly, the recruitment of ALYREF export factors to viral mRNAs by ORF57 inhibited the interaction of viral mRNAs with hMTR4, thus protecting these transcripts from degradation (86). Notably, in the absence of ORF57 in KHSV-infected epithelial cells (HeLa cell line), viral transcripts were found to be directed to degradation via the poly-A-binding protein nuclear 1 (PABPN1) and PAPa/y pathway, both host degradation pathways for polyadenylated nuclear transcripts (86). In this pathway, host mRNA degradation factors were recruited to the poly-A tail of the mRNAs to promote their degradation via arsenic resistance protein 2 (ARS-2), which is a 5′-CAP end-dependent degradation pathway (Table 1) (Fig. 2B) (86).

Overall, ICP27 homologs can have direct effects on the efficiency of translational of viral mRNAs, a role that is somewhat conserved among several herpesviruses, namely, HSV-1, EBV, and KHSV, yet it remains to be studied in HSV-2, VZV, HCMV, HHV-6, and HHV-7. Notably, this role, when described, has been reported to occur through direct interactions with polyribosomes, as described for ICP27 of HSV-1, or through interactions with key host proteins related to the initiation of translation, as reported for EB2 of EBV and ORF57 of KSHV.

## OTHER ROLES OF ICP27 HOMOLOGS: REGULATION OF HOST INTERFERON RESPONSES AND VIRION RELEASE

Besides the many roles described above for ICP27 and its homologs in herpesviruses, which involve mRNA transcription, processing, export, and translation, additional roles have also been reported for these proteins, evidencing their multifaceted nature.

One such novel role described for ICP27 of HSV-1 involves the regulation of type-I interferon (IFN-I) responses. Infection with HSV-1 strains KOS, 17+, or McKrae is characterized by significant decreases in type-I IFN production at the protein level, 12 and 18 hours post-infection in the monocyte cell line THP-1 cells when compared to the HSV-1 F strain (56). However, if these cells are infected with a virus lacking ICP27 in the KOS background, it was found that the inhibition of type-I IFNs was significantly reversed at 12 hours post-infection. The same authors studying this phenomenon now in epithelial cells (HEK-293T cell line) found that the possible mechanism by which ICP27 interferes with the cell type-I IFN response is through the inhibition of the expression of the stimulator of interferon genes protein (STING) within the cyclic GMP-AMP synthase (cGAS) (cGAS-STING) pathway. Consistently, when performing the experiment described above, but in cells with STING deleted, the previously reported effects of HSV-1 over the production of type-I IFNs in the cells infected with the ICP27 mutant virus were reversed (56). This finding allowed us to conclude that ICP27 interferes with the c-GAS/STING signaling pathway by inhibiting the phosphorylation of TANK-binding kinase 1 (TBK-1), which overall results in the inhibition of the transcription of type-I IFN genes in monocytes (THP-1 cell line). Site-directed mutation experiments in the same study concluded that the most relevant domain of ICP27 involved in the modulation of the type-I IFN response was the RGG domain (56). However, the mechanism by which this RGG domain interferes with IFN-I production remains to be determined.

The VZV ICP27 homolog IE4 has also been studied in other contexts apart from those related to interactions with RNA molecules, namely, in the context of latency, with current evidence indicating that IE4 can be found at both the RNA and protein levels in VZV-latently infected human lymph nodes, suggesting that the expression of this factor continues to occur during periods of latency (48). To further study the role of IE4 during VZV latency, cotton rats were infected with a VZV lacking IE4, or VZV in which IE4 was reintroduced (rescue virus). While 100% of cotton rats inoculated with the virus lacking IE4 or the rescue virus were positive for VZV, when dorsal root ganglia were extracted 6 weeks post-inoculation, it could be evidenced that the group inoculated with the virus lacking IE4 displayed reduced frequency of latency and less abundance of ORF63 mRNA (a transcript associated with latency in VZV) than the rescued virus, suggesting IE4 has a role in maintaining latency (48). Given this result, the role of ICP27 homologs in the context of latency seems to be an interesting area to investigate. It is important to note that it has not been determined whether this protein is directly or indirectly involved in latency and which other ICP27 homologs may share similar properties as IE4 in this regard (48).

Another function reported for ICP27, particularly the homolog encoded within HSV-2, is related to virion release. Park and colleagues generated an ICP27-mutant HSV-1 expressing ICP27 of HSV-2, which was seemingly as functional as the wild-type virus in epithelial cells (Vero cells) (88). However, an effect resulting from this modification was related to the morphology of the viral plaques generated by the mutant virus, with the latter generating round plaques, while the WT virus generated comet-shaped plaques in liquid medium overlay containing 1% normal pooled human serum (PHS) previous to staining with Giemsa (88). Given these results, the authors suggested that the comet-shaped plaques were likely due to secondary infections mediated by infective particles present in the culture medium and convective flow, but more importantly, the observed results suggested that ICP27 of HSV-2 has a role in virion release because mutant viruses were more efficiently retained on the cell surface compared to the WT virus. However, the mechanism by which ICP27 would mediate this role is unknown and is an exciting question to address (88).

Altogether, these findings argue for additional roles for ICP27 homologs beyond direct interactions with RNA molecules. Yet, they have only been evidenced so far in a limited number of herpesviruses. Thus, assessing whether these effects expand onto other viruses within the family will be important. Furthermore, it will be relevant to determine the mechanisms related to the observed effects and whether these could have potential therapeutic implications in future drug development.

## CONCLUDING REMARKS

Herpesviruses encode numerous gene products that modulate key cellular processes to facilitate their replication and perpetuation. Among these, ICP27 of HSV-1 and its homologs emerge as key viral determinants participating in numerous central processes related to the biology of mRNAs, impacting relevant processes, such as gene transcription, splicing of mRNAs, their export to the cytoplasm, and mRNA translation. Additionally, current evidence points out the significant effects of these proteins over host mRNAs, which most likely is oriented at favoring the translation of viral mRNAs into the corresponding proteins. However, the functions and molecular mechanisms related to the modulation of cellular processes by ICP27 and its homologs have been somewhat understudied for some herpesviruses, namely, for HHV-6 and HHV-7, which deserves more attention to identify potential common features between these herpesvirus proteins and a potentially common treatment between them. Given the relevance of the numerous processes carried out and modulated by ICP27 and the corresponding homologs during herpesvirus infections, these proteins emerge as relevant pharmacological targets for reducing virus replication and hopefully, the diseases they elicit. Nevertheless, based on the large number of domains that these proteins encode, it may be necessary to identify drug cocktails that simultaneously target several functional regions to more broadly inhibit their functions. Most importantly, these drugs remain to be identified or developed as they are currently inexistent, yet with the current advances in structural biology supported by machine learning, we anticipate these drugs could be available in the near future.
